# Measuring the efficiency of Palestinian public hospitals during 2010–2015: an application of a two-stage DEA method

**DOI:** 10.1186/s12913-018-3228-1

**Published:** 2018-05-29

**Authors:** Wasim I. M. Sultan, José Crispim

**Affiliations:** 1 School of Economics and Management, University of Minho, 4710-057 Braga, Portugal; 2 P.O. Box 198, Hebron, Palestine

**Keywords:** Public hospitals, Efficiency, 2-DEA, Tobit regression, West Bank

## Abstract

**Background:**

While health needs and expenditure in the Occupied Palestinian Territories (OPT) are growing, the international donations are declining and the economic situation is worsening. The purpose of this paper is twofold, to evaluate the productive efficiency of public hospitals in West Bank and to study contextual factors contributing to efficiency differences.

**Methods:**

This study examined technical efficiency among 11 public hospitals in West Bank from 2010 through 2015 targeting a total of 66 observations. Nationally representative data were extracted from the official annual health reports. We applied input-oriented Data Envelopment Analysis (DEA) models to estimate efficiency scores. To elaborate further on performance, we used Tobit regression to identify contextual factors whose impact on inefficient performance is statistically significant.

**Results:**

Despite the increase in efficiency mean scores by 4% from 2010 to 2015, findings show potential savings of 14.5% of resource consumption without reducing the volume of the provided services. The significant Tobit model showed four predictors explaining the inefficient performance of a hospital (*p* <  0.01) are: bed occupancy rate (BOR); the outpatient-inpatient ratio (OPIPR); hospital’s size (SIZE); and the availability of primary healthcare centers within the hospital’s catchment area (PRC). There is a strong effect of OPIPR on efficiency differences between hospitals: A one unit increase in OPIPR will lead a decrease of 19.7% in the predicted inefficiency level holding all other factors constant.

**Conclusion:**

To date, no previous studies have examined the efficiency of public hospitals in the OPT. Our work identified their efficiency levels for potential improvements and the determinants of efficient performance. Based on the measurement of efficiency, the generated information may guide hospitals’ managers, policymakers, and international donors improving the performance of the main national healthcare provider. The scope of this study is limited to public hospitals in West Bank. For a better understanding of the Palestinian market, further research on private hospitals and hospitals in Gaza Strip will be useful.

**Electronic supplementary material:**

The online version of this article (10.1186/s12913-018-3228-1) contains supplementary material, which is available to authorized users.

## Background

The healthcare system in the Occupied Palestinian Territories (OPT) is influenced by the ambiguous political environment within which it is enacting [1]. The OPT (West Bank, East Jerusalem, and Gaza Strip) is a country in chronic conflict and economic emergency [2]. The never-ending conflict between the Palestinians and the Israelis seemed to come to an end when the Middle East peace process was settled, particularly, after the Madrid conference in 1991, then the Oslo Accords in 1993 and the establishment of the Palestinian Authority (PA) in 1994. Henceforth, building the capacity of the Palestinian public healthcare sector evolved [3], and had undergone several reforms. Reforms were heavily subsidized by international donations [4], as efforts made by the international community to resolve the conflict in Palestine-Israel through economic encouragements [5].

Despite the noticeable progress in rebuilding the institutions of the yet to be “The State of Palestine,” ground reality suggests otherwise. The situation is remaining complicated and problematic as witnessed by more isolation and more restrictions on movement between West Bank (WB) and Gaza Strip (GS) and between cities within WB. The Palestinians are not allowed to travel freely between the OPT regions [1]. To date, the Israelis control over water, electricity, borders, and transport amongst other infrastructural matters, while, the Palestinians have limited control over their own affairs. This unique context has implications on the priority settings and the process of health policy implementation [6]. Therefore, in practice, the integration of health policies and health delivery operations is not just a matter of combining the two.

The Palestinian Ministry of Health is the leading healthcare provider including hospital care and bears the most substantial burden to meet the constant growth in the demand for healthcare services. On average, health expenditure recorded consistent annual growth rate of 7%. The total health expenditure increased from $400 million in the year 2000 to $1400 million in the year 2015; the latter accounted for 10.7% of the country’s Gross Domestic Product (GDP). The public reimbursement schemes represent 62.5% of the total health expenditures [7].

Hospitals, with 6006 beds, are the main healthcare providers to serve 4.48 million people living in WB and GS. Forty-two percent of the total expenditure is spent on hospital care (i.e., 4.5% of the country’s GDP) [8]. Because hospitals make up a large portion of healthcare expenditure, hospitals are a potentially large source of cost savings. Therefore, the analysis of this study was intended to capture potential gains in the efficiency of public hospitals that may have a substantial contribution to large potential cost-savings of the country’s healthcare expenditure [9–11].

Moreover, the applied governmental health insurance scheme covers most of the Palestinians, by which they are entitled to public services, had increased the burden on public hospitals. Therefore, public hospitals (61.1% of all the hospital beds) are crowded and functioning at high bed occupancy rates or even over occupied [2]. To cater to the increasing health demand on healthcare services, the Palestinian Ministry of Health (PMoH) allocates about 40% of its budget to purchase hospital services from other referral hospitals within the country or abroad such as hospitals in Jordan [12]. Recently, the World Health Organization (WHO) report indicated that the decline in donors’ support and the unique political situation of the Palestinians have serious effects on the scope and quality of health conditions [13].

The purpose of this work is twofold, analyzing the efficiency of the public hospitals in West Bank; and evaluating the environmental factors affecting their productivity. Keeping in mind that the hospital technical efficiency requires the use of minimum input to produce a given level of output [10] and that the ability of a hospital to transform inputs into outputs is influenced by its managerial efficiency as well as the external operating environment [14, 15].

The scope of our work is limited to public hospitals in West Bank. Hospitals in Gaza Strip are excluded in this work due to many limitations: (1) The geographical separation between West Bank and Gaza, the Palestinians are not allowed to travel across them; (2) The 2008 and 2014 wars against Gaza makes the context of hospital operations incomparable; (3) The Palestinian internal conflict since 2006 escalated with the split of Palestinian Authority into one government in WB and another in GS, hence, the operational data of hospitals in GS is unreliable. Therefore, the main scope of this papers is to examine the technical efficiency of 11 public hospitals out of 13 public hospitals working in West Bank during 2010–2015 (i.e., 66 observations).

We conducted secondary research to find studies evaluating the performance of healthcare providers in Palestine; to date, there are no previous studies concerning the topic. The existing relevant literature describes the transitional context and the complications within the country’s healthcare system in Palestine [2–4, 6, 16–19]. Hence, improving performance among the Palestinian public hospitals by performance measurement is a straightforward need. The generated information will provide valuable insights to hospital managers who make operational decisions and to policymakers and international donors who may influence the external operating environment by regulations, subsidies or by other policy measures.

Data Envelopment Analysis (DEA) is a universal methodology in healthcare evaluation and widely used non-parametric methodology to evaluate performance [20–22]. Since the advent of DEA by Charnes et al. [23], more than 10 thousand studies had been published which estimated the performance of different kinds of entities and production activities including the healthcare sector [24]. Recent DEA studies extend the analysis to investigate variations in hospital performance over years and to identify contextual drivers of efficient practices [25].

Due to the lack of data in developing countries, few empirical works applied the data-based methodology of DEA models [26]. To date, no studies have examined the performance of public hospitals in Palestine for potential improvements. Therefore, this work addresses a DEA literature gap by analyzing the efficiency of the public hospitals and identifying contextual drivers of inefficient performance in a developing country, namely, Palestine. In response to this need, our endeavor goes to achieve the following research objectives: (1) evaluate how Palestinian public hospitals utilize resources while caring for their patients from 2010 to 2015; and (2) explore environmental effects associated with the efficient use of hospital resources.

We apply two-stage data envelopment analysis (2-DEA) where the efficient frontier and the hospital level efficiency score are estimated with DEA model in the first stage, and the efficiency estimates are regressed on contextual factors in the second stage [15, 27]. In stage 1, we calculate the efficiency with which physical inputs produce output. In stage 2, we apply Tobit regression which is commonly used to relate efficiency scores to factors expected to influence efficiency while these factors are not under the control of hospital managers [14, 28, 29].

### Empirical context

The whole area of the OPTs is 6170 km^2^ of which 5800 km^2^ is the area of WB, and 365 km^2^ is the area of GS. The Palestinian healthcare system comprises five main providers of healthcare services: (1) The Palestinian Ministry of Health (PMoH) and represents the public sector; this sector comprises primary healthcare centers and public hospitals. These hospitals are owned and administered by the Palestinian Ministry of Health. They are general hospitals that provide primary and secondary healthcare services, however; no public hospital provides tertiary services. (2) The United Nations Relief and Works Agency for Palestine Refugees (UNRWA); (3) Non-Governmental Organizations (NGOs); (4) Palestinian Military Medical Services (PMMS); and (5) Private for-profit organizations. According to the Palestinian Central Bureau of Statistics (PCBS), these providers manage80 hospitals with a capacity of 6006 hospital beds to serve 4.88 million people living in OPT, of which 2.97 million are living in WB, and 1.91 million are living in GS. The median age of the Palestinians is 19.8 years, and 39.4% of the population is under 15 years old. The age group (0–4 years) is 15% while for the age group over 65 years constitute only 2.9% of the population [12, 30].

There are 50 hospitals are operating in WB including East Jerusalem (60.1% of total beds), and 30 hospitals are operating in GS (39.9% of all the beds). 73% of all the hospital beds are general beds, 19% are specialized beds, 3.1% rehabilitation are beds and 4.9% are maternity beds (Table 1).

**Table 1:** Distribution of hospital beds and primary healthcare centers in OPTs in 2016

Hospitalization					
Type of hospitalization	Regions	Public^a^	Others^b^	By region	Hospital beds
General	WB	1414 (32.2%)	1222 (28%)	2636 (60.2%)	
	GS	1328 (30.2%)	421 (9.6%)	1749 (39.8%)	4385 (73%)
Specialized	WB	180 (15.7%)	437 (38.2%)	617 (53.9%)	
	GS	293 (25.6%)	234 (20.5%)	527 (46.1%)	1144 (19%)
Rehabilitation	WB	0.0	141 (76.2%)	141 (76.2%)	
	GS	0.0	44 (23.8%)	44 (23.8%)	185 (3.1%)
Maternity	WB	0.0	213 (72.9%)	213 (72.9%)	
	GS	43 (14.7%)	36 (12.4%)	79 (27.1%)	292 (4.9%)
Total by region	WB	1594 (44.2%)	2013 (55.8%)	3607 (60%)	
	GS	1664 (69.4%)	735 (30.6%)	2399 (40%)	
Total beds		3258 (54.3%)	2748 (45.7%)	6006 (100%)	6006 (100%)
Public Primary Care Centers (PHCs)					
	WB	422 (69.4%)	186 (30.6%)	608 (80.0%)	
	GS	49 (32.2%)	103 (67.8%)	152 (20.0%)	
Total PHC		471 (62.0%)	289 (38.0%)	760 (100%)	

Public^a^, hospitals or PHCs are owned and administered by the Palestinian Ministry of Health

Others^b^, hospitals or PHCs are not owned nor administered by the Palestinian Ministry of Health

In West Bank, public hospitals are distributed in 11 administrative areas (governorates). They are Jenin, Tubas, Tulkarm, Nablus, Qalqillya, Salfit, Ramallah, Jericho, Bethlehem, Hebron, and East Jerusalem. However, due to political reasons, there is no Palestinian public hospital in East Jerusalem. Therefore, the included hospitals in this study are 13 public hospitals with a capacity of 1594 beds working in WB. To have a homogeneous sample of general hospitals, we excluded two hospitals from the analysis: a new hospital with 37 beds was established in 2014 (P12 in Table 2), data is not available from 2010 to 2013; the other hospital is psychiatric with 180 beds. As a result, we analyze the efficiency of 11 public hospitals (P01-P11 in Table 2) from 2010 to 2015 (66 observations). Table 2 illustrates the sample characteristics and relevant market attributes during 2015.

**Table 2:** Selected characteristics of the operating public hospitals in West Bank (2015)

Governorate	Market characteristics			Public hospital characteristics			
	PHC/10000	Beds/10000	% public beds	Public hospitals	Beds	Occupancy rate	Hosp.
Hebron	2.22	9.0	48.9	Abu al Hasan	36	101.3	P01
Salfit	4.08	7.1	100.0	Yasser Arafat	50	71.9	P02
Jericho	3.85	10.4	56.4	Jericho	54	71.7	P03
Nablus	1.84	16.9	40.8	Watani	55	86.0	P04
Qalqilya	3.51	10.9	47.9	D. Nazal	58	95.0	P05
Tulkarm	2.36	9.3	69.1	Thabit Thabit	117	71.5	P06
Bethlehem	2.04	27.3	22.2	Al Hussein	131	79.4	P07
Jenin	2.09	7.1	73.8	Khaleel S.	163	90.1	P08
Nablus	1.84	16.9	40.8	Rafedia	200	87.6	P09
Ramallah	2.18	12.2	56.1	Med. Complex	238	97.6	P10
Hebron	2.22	9.0	48.9	Alia	275	120.4	P11
Tubas	–	5.7	–	The Turkish	37	63.1	P12^a^

*PHC* Primary Health Care Centers

^a^Hospital P12 is excluded, available data is limited to 2014 and 2015

### Production model and variables

**Fig. 1:**
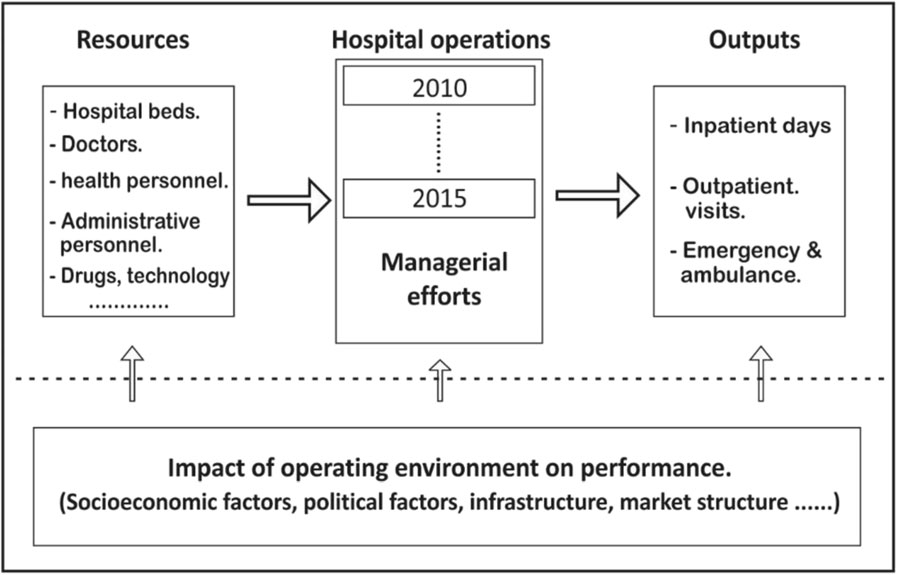
Conceptual production structure of hospitals

The ability of a hospital to transform inputs into outputs is influenced by its managerial efficiency (practices) and external operating environment (operational conditions) [15]. Therefore, relating the measures of inefficiency to the surrounding contextual factors provides a better understanding of efficiency differences and determines the key performance drivers across hospitals [31]. The OPT has a fragmented landscape of healthcare providers including hospitals which evolved across different regimes [1, 4]. However, the geopolitical setting of the OPT poses challenges to healthcare delivery and access, therefore, it is believed that environmental factors touch the production of healthcare services and should be included in our analysis. Figure 1 shows the relationships between input-output measures and contextual factors.

Different input and output sets had been used in the DEA literature to analyze the efficiency of hospitals [32, 33]. The basic principle, to identify variables, is to have a clear understanding of the “process” being evaluated among peer hospitals [34]. The investigated hospitals are all general hospitals; they are designed to provide primary and secondary health services, they don’t provide tertiary health services. Therefore, we included input-output measures that make a practical sense for the Palestinian public hospital settings. We used output measures that represent the level of public health benefits achieved in respect of three functional areas; admissions, outpatient visits, and emergency services. Since the other activities within the hospital (e.g., laboratory tests, deliveries, surgical operations, radiology activities) are highly correlated with the three measures, we did not include them in the set of outputs [35].

We included three output measures, they are: (1) inpatient services as measured by the total number of annual care days rather than a number of cases to account for case-mix adjustment [36]; (2) outpatient services as measured by the total number of annual visits [33]; and (3) the emergency services as measured by the total annual number of cases served without admission [37].

Inpatient days represent the total annual duration of patient admissions and the utilization of clinical and nonclinical inputs, such as nursing care, pharmaceutical items, paramedical support services, and administrative services. Outpatient visits represent the utilization of the outpatient clinics and the dedicated clinical and administrative resources to these clinics. In Palestine, the emergency departments and the ambulance services are vital outputs and represent the utilization of a considerable amount of resources in the public hospitals. The reasons behind the imperative role of emergency services are: (1) the hospital emergency departments become the first choice for patients seeking treatment because family practice model is absent in Palestine; (2) the primary healthcare centers work only for 6 hours a day, and 5 days a week, they provide a minor role of emergency and ambulance services; and (3) the majority of the population is covered by the government health insurance scheme by which they are entitled to the emergency departments in public hospitals [2].

In line with other DEA literature [38–40], we included four input measures. They characterize the employed labor and capital. Labour input measures comprise three groups of personnel, the doctors, the healthcare full-time employees FTEs (e.g., Nurses, technicians, and other employees in para-medical departments), and the administrative FTEs [41]. Capital input measure was represented by the number of hospital beds [42]. Data on other resources, such as drugs, laboratory tests, or instruments were not available for the included hospitals.

As for the impact of the environment on the productivity of the public hospitals in West Bank, we considered ten factors (Table 3). These factors are organized into three sets: (1) Factors had been previously studied by other researchers such as the bed occupancy rate (BOR) and the average length of stay (ALOS). (2) Factors represent some proposed market settings in Palestine such as the percentage of public hospital beds (PPHB) and the availability of primary healthcare centers in the governorate where the included hospital serves (PRC). (3) Factors concerning the unique context of WB such as the percentage of refugees living in the governorate (REFP) where the included public hospital serves.

**Table 3:** Potential contextual factors

Variable	Definition	Measurement	Mean^a^	SD
BOR	Bed occupancy rate	The proportion of occupied beds in a year = Inpatient days / (number of beds ^a^ 365).	83.1%	1.65%
OPIPR	Outpatient – inpatient ratio	Total outpatient visits divided by total inpatient days.	1.23	0.06
ALOS	The average length of stay	Total inpatient days divided by the number of admissions.	2.18	0.05
ADHR	The ratio of administrative to health employees.	The number of administrative FTEs divided by the total health FTEs including doctors in each observed hospital.	0.36	0.01
SIZE	Hospital size (dummy)	(1) For large hospitals > 130 beds, (0) otherwise.	0.36	0.06
LOC	Hospital location (dummy)	(1) for North Governorate and (0) for South Governorate.	0.6	0.06
REFP	The proportion of refugees living in the governorate.	The percentage of refugees living in camps of all the governate population where the observed hospital operates.	8%	0.8%
HPFP	Number of hospital beds per 10,000 inhabitants	The number of all the available hospital beds per 10,000 in the governorate where the public hospital operates.	12	0.711
PRC	The available primary care centers per 10,000 inhabitants	The number of primary centers per 10,000 inhabitants in the governorate where the public hospital operates.	2.7	0.1
PPHB	The percentage of public hospital beds.	The percentage of the available public hospital beds in a governorate to the total available number of beds.	59.3%	2.8%

^a^Mean and SD Values used six-year data of the predictors from 2010 to 2015

Data Source: Palestinian annual health reports 2010–2015

As for the first set, six factors are included. (1) The bed occupancy rate (BOR) is related to return to scale within hospital operations and capacity utilization, the higher the BOR, the higher constant return to scale and scale efficiency [40]. From economic point view, higher occupancy rate has a lower cost per case [43]. (2) The ratio of outpatient visits to inpatient days (OPIPR) shows to what extent hospital managers make a better combination of the two services that could make better use of available resources. (3) The average length of stay (ALOS) is the average days spent in a hospital from the time of admission to the time of discharge. It represents the intensity and efficiency by which individual patients are treated [22]. (4) The ratio of administrative employees to health employees including doctors (ADHR) may affect the way of doing clinical and nonclinical processes during hospitalization, accordingly may influence efficiency [39]. (5) The size (SIZE) of the hospital and the applied processes to patient treatment may differ as for their size and affect the level of resource utilization; a large hospital may suffer diseconomies of scale [44]. (6) Although all the investigated hospitals are public and don’t compete, it was felt that the market characteristics of each region may impact efficiency [33]. The proposed factors influence patients’ choices and may influence hospital efficiency. Due to differences in demographic and socioeconomic factors, we considered the location of the hospital (LOC) as a dummy variable to indicate whether the hospital is North to Jerusalem or South to Jerusalem where different social lifestyles apply.

As for the second set of environmental factors, two concentration indicators as a proxy for provider distribution were included: (7) The available number of primary health centers per 10,000 citizens in each governorate (PRC). (8) The percentage of public hospital beds (PPHB) to the overall providers’ beds in a certain governorate [45].

As for the third set of environmental factors, additional two factors are included. They apply to the unique context of Palestine. (9) Since the Palestinians’ loss of their land and homes in 1948, tens of thousands of Palestinians were displaced to live in refugee camps in West Bank cities; this factor was thought to influence efficiency; the way how refugees are living and working may influence the efficiency of the working hospital in that governorate. Therefore, the percentage of refugees living in every governorate (REFP) was considered. (10) The Palestinian healthcare system comprises fragmented healthcare providers evolved through different regimes, the number of available hospital beds per 10,000 inhabitants in a given administrative area was considered It represents the supply side of hospital services in a governorate (HBFP). Table 3 displays the ten proposed environmental factors.

### Two-stage data envelopment analysis (2-DEA)

The problem of measuring productive efficiency was best described, 60 years ago, by Farrell [46]. To solve the problem, Farrell introduced an activity analysis approach that combines the measurement of multiple inputs into a single measure of efficiency which he regarded as “technical efficiency.” Technical inefficiency is the amount of waste that can be eliminated without worsening any input or output. Building on Farrell’s ideas, Charnes et al. [23] introduced a powerful nonparametric methodology to assess the relative efficiencies of multi-input and multi-output production units such as hospitals which had been titled Data Envelopment Analysis [47]. These production units are denoted as decision-making units (DMUs) in the DEA literature.

The first published DEA work in healthcare context was in 1983 and investigated nursing services [48]. In 1984 the second published study investigated the medical and surgical departments in seven hospitals [49]. Among the empirical studies using DEA, hospitals received the most research attention [50]. The goals of hospital services are multiple and complex. Hospitals produce multiple outputs (e.g., inpatient care, surgeries, outpatient care, emergency) and absorb multiple inputs (e.g., clinical and non-clinical staff, beds, equipment, and supplies).

Based on a review of 317 published studies on frontier measurement of the efficiency of the healthcare delivery from1983 to 2006, Hollingsworth [51] found that 75% of the works applied the DEA, and other DEA–based methods. Empirical applications of DEA included performance examinations of different healthcare markets ranging from primary healthcare level [20, 52] to home healthcare agencies [53] and hospitals [54]. And from practice behavior at provider group level was also examined [55, 56] to the overall healthcare system and country level [45, 57].

The two-stage DEA is commonly used in productive efficiency analysis to estimate the impact of environmental factors and practices on performance. Because the DEA efficiency estimates of the first stage represent censored data, the second stage of analysis applies Tobit regression [28, 33]. Tobit regression applies the Maximum Likelihood Estimator (MLE) to find the model’s parameters [58]. The second stage generates additional information on managerial performance if we filter the impact of the component associated with the contextual factors. Further, the second stage analysis informs policymakers who may influence the operating environment [14, 15].

Many studies used the DEA efficiency score in the second stage analysis to evaluate the influence of operating environment on efficiency. Chowdhury & Zelenyuk [42] applied DEA and truncated regression model to explore the determinants of the hospital efficiency in Ontario/Canada. Their findings identified occupancy rate, outpatient-inpatient ratio, location, teaching status, and case-mix index as determinants of efficient practices. A study examined the hospitals in Ghana used DEA and Tobit regression, efficiency was determined by region and ownership [59]. Finally, Samut & Cafrı [45] analyzed the healthcare systems in 29 OECD countries during 2000–2010 and applied Malmquist Index and Tobit regression procedures, The authors, identified education, income, and market factors as determinants of hospital efficiency.

Despite the extensive body of DEA literature examining the performance of healthcare sector at all levels, due to the scarcity of data, few empirical studies were conducted in developing countries. Most DEA works were applied in the developed countries, mainly the US and Europe [60]. Particularly, in Arabic Speaking Countries, two previous studies employed the DEA and investigated the efficiency of hospitals in Jordan and Sultanate of Oman [38, 61]. Aimed at Palestine, to date, no studies have examined the efficiency of Palestinian hospitals or the influencing contextual factors. The performance measurement systems are already absent within the country’s healthcare organizations.

## Methods

This work addresses the productive efficiency of the Palestinian public hospitals from 2010 to 2015. We extracted the relevant operational data from the published Annual Health Reports by the Palestinian Ministry of Health (PMoH). To achieve our research objectives, we organized the analysis around two key steps: (1) Using a six-year data of the Palestinian public hospitals, we employ the basic DEA-CCR and the DEA-BCC models to analyze the overall efficiency, pure technical efficiency and scale efficiency; (2) we regress the DEA-CCR scores of 66 observations of the first step on ten potential contextual factors. We apply Tobit regression to find the factors whose impact on efficiency is statistically significant.

### Sample and data

The study used data from 11 public hospitals operating in West Bank from 2010 to 2015 targeting a total of 66 observations. The sample excluded two public hospitals from the analysis. One psychiatric hospital in Bethlehem (180 beds) does not meet the homogeneity assumption of DEA method. Another newly established hospital in 2014 (37 beds) was also excluded because efficiency judgment of a new hospital could be biased in the early stages of managerial experience. We obtained ethical approval from the Palestinian Ministry of Health (PMoH) to carry out the research.

The investigated hospitals (1377 beds) are owned and administered by the Palestinian Ministry of Health. They are general hospitals and their resources are assigned from the ministry based on requests from their managers. Their patients are coved by a governmental insurance scheme by which patients are entitled to public hospitals. Then, patients are treated within the public hospital under two conditions; the availability of the required clinical services and the availability of unoccupied hospital bed, otherwise, the patient is transferred to other provider and financially covered by the applied insurance scheme. Hospital managers are asked to manage the given demand while managing the hospitals’ resources accordingly.

Public hospitals in WB are geographically distributed across ten governorates (see Table 2); one public hospital serves one governate. Hebron and Nablus are two exceptions where two hospitals serve in each governorate. Data on four input measures and three output measures have been extracted from the Annual Statistical Healthcare Reports published by the PMoH. Table 4 illustrates the year-specific means and standard deviations of the included input-output measures.

**Table 4:** Distribution of input-output measures, means and standard deviations, *N* = 11

Year	Input measures				Output measures		
	Hospital beds (X1)	Doctors FTEs (X2)	Health FTEs (X3)	Administrative FTEs (X4)	Inpatient days (Y1)	Outpatient visits (Y2)	Emergency care (Y3)
2010	107	55	164	74	32,152	38,111	56,082
	(20)	(8)	(28)	(9)	(6667)	(7702)	(7452)
2011	106	46	170	75	32,101	35,085	56,872
	(19)	(8)	(29)	(9)	(6728)	(7491)	(7915)
2012	111	47	174	75	36,015	41,305	65,094
	(21)	(7)	(29)	(8)	(7914)	(8430)	(9461)
2013	119	44	179	75	37,719	40,983	66,301
	(23)	(6)	(27)	(7)	(8678)	(8117)	(11292)
2014	123	46	195	77	39,908	41,737	69,016
	(25)	(6)	(32)	(8)	(9837)	(8652)	(11284)
2015	125	49	194	74	42,692	46,017	68,425
	(25)	(7)	(32)	(8)	(10588)	(8994)	(10272)

*FTEs* Full-Time Employees. Health FTEs, medical personnel other than doctors, such as nurses, laboratory technicians, and radiology technicians

### Estimation of productive efficiency

We employ two milestones DEA models, namely the CCR [23] and the BCC [62]. The letters in “CCR” and “BCC” stand for the initials of the developers’ last names. These two models have become standards in the literature of performance measurement under the assumptions of constant and variable returns to scale respectively [63]. Because public hospitals serve the public demand as given and must manage their resources accordingly, therefore, they target input minimizing rather than output maximization which recommends using the input-oriented DEA models [35, 64–66]. We address the potential input savings and constructs input-oriented frontiers guided by the space of managers’ control.

First, we applied a DEA-CCR model which assumes a Constant Returns to Scale (CRS) within hospital operations and doesn’t account for the scale effects; then, we applied the DEA-BCC model which was developed in 1984 to satisfy scale effects in efficiency analysis. The mathematical formulation CCR dual linear programming model to estimate relative efficiencies of 11 hospitals is written as the following linear problem:

1 $$ {\theta}_o^{\ast }=\mathit{\operatorname{Min}}\;{\theta}_o $$

Subject to,

$$ {\displaystyle \begin{array}{cc}{\sum}_{p=01}^{p=11}{\lambda}_p{x}_{ip}\le \kern0.62em \theta {x}_{io}& i=1,2,3,4\\ {}{\sum}_{p=01}^{p=11}{\lambda}_p{y}_{rp}\ge \kern0.62em {y}_{ro}& r=1,2,3\\ {}{\lambda}_p\ge 0& p=1,2\kern0.5em ..,11\end{array}} $$

Where:

θ_o_ = the efficiency score of hospital “0” under evaluation.

x_ip_ = the quantity of input “i” utilized by the “p^th^” hospital.

y_rp_ = the quantity of output “r” produced by the “p^th^” hospital.

λ = weights obtained from the dual version of the linear programming.

The radial distance to frontier provides a technical efficiency measure for hospitals under assessment. The DEA-BCC input-oriented model requires an additional set of convexity constraint for the dual linear programming algorithm (Eq. 1), the sum of lambdas to be one and written as Eq. 2:

2 $$ {\sum}_{p=01}^{p=11}{\lambda}_p=1.0 $$

The sum of lambdas yielded from the CCR model provides information whether the hospital is operating under increasing or decreasing returns to scale [67, 68]. While the CCR efficient hospitals are operating at the most productive scale size and the sum of lambdas is one, the inefficient hospitals are operating under Decreasing Returns to Scale (DRS) when **∑λ** > 1 and may benefit from economies of scale. Other inefficient hospitals are operating under Increasing Returns to Scale (IRS) when **∑**λ < 1 and may suffer diseconomies of scale that may explain a state of weak control among large hospitals.

Since the BCC model always envelops the data more closely than the CCR model (input-oriented frontiers). Inefficient hospitals measure the shorter distance to the BCC frontier than the CCR frontier [69]. The analysis of the two models distinguishes three types of efficiencies that help managers to capture the components of inefficient operations [70, 71]. They are global technical efficiency (TE) as given by CCR score, pure technical efficiency (PTE) as given by the BCC score, and scale efficiency (SE) reflects the portion of inefficiency attributed to the given scale of operations (Eq. 3):

3 $$ {\displaystyle \begin{array}{c} CCR\; score= BC\mathrm{C}\; score\times Scale\kern0.5em efficiency\\ {} TE= PTE\times SE\end{array}} $$

**Fig. 2:**
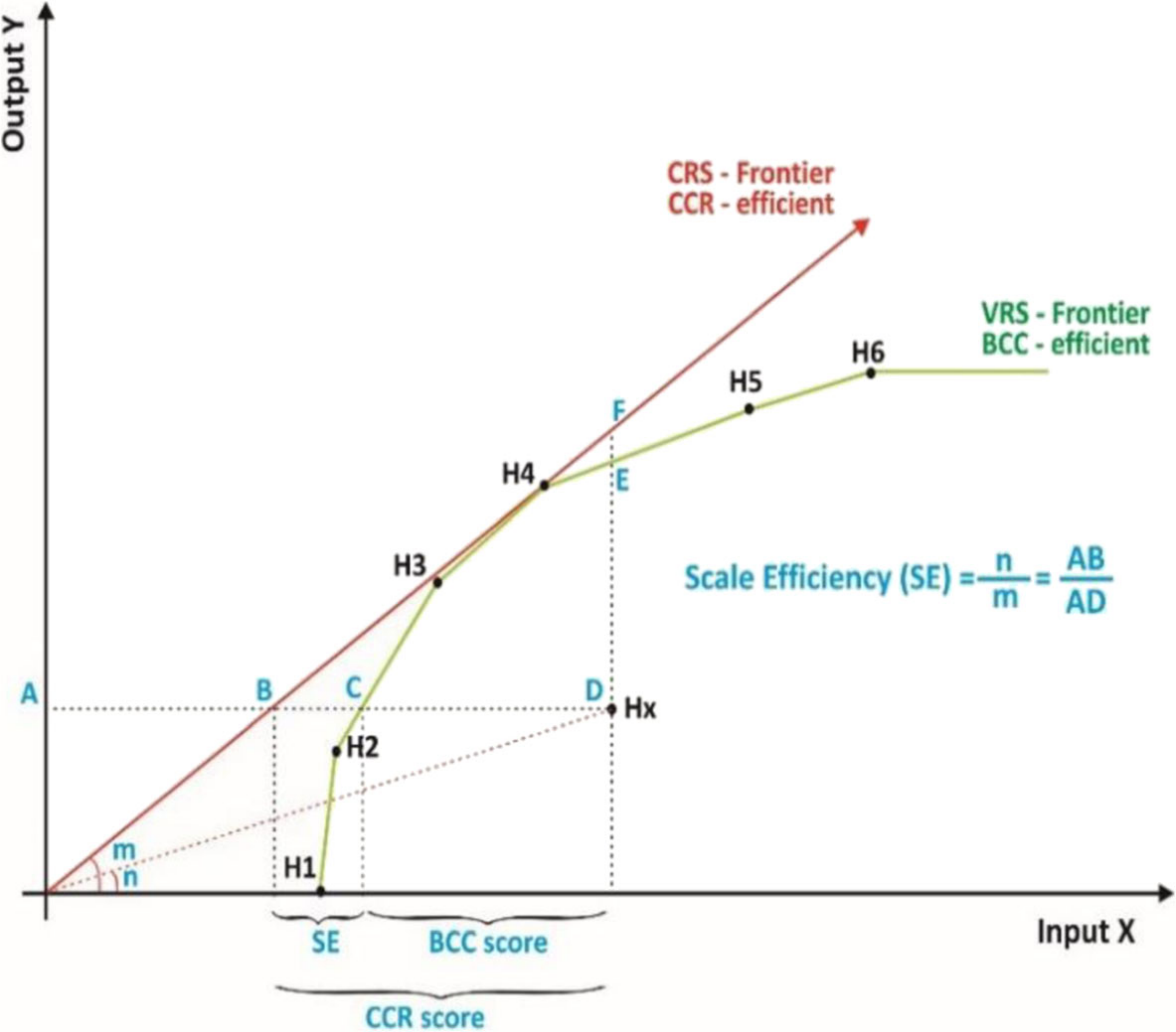
Illustration of SE derived from the CCR scores and the BCC scores. Reproduced from Banker et al. [62]

Reproducing the graph of Banker et al., [62], Fig. 2 illustrates the application of the CCR and BCC scores regarding the three components of efficiency related to the proposed production possibility set for the input-output mix (X, Y). Group of Hospitals “H1 to H6 and Hx” were used for demonstration purpose. The inefficiency component of hospital Hx as given by the ratio AB/AD is attributed to the scale of its operations. Moreover, it is distinguished from the pure technical inefficiency as given by the ratio AC/AD.

Because it is important to have a sufficient number of observations we employed the DEA framework presented by Boussofiane et al. [71]. The method allows us to capture the actual variations of each hospital through simultaneous estimation of efficiency of all the 66 observations (*N* = 66). This method strengthens the discriminatory power of DEA as sufficient number of DMUs are analyzed [34].

DEA is a relative measurement method, a change in the efficiency score in the following year of the tested hospital does not necessarily mean a change in its performance only; changes in the performance of the others may influence the relative position of that hospital. If we carry out an independent analysis for each year, we cannot certainly attribute the changes in the efficiency score of a focal hospital to actual performance change of that hospital. But, simultaneous inclusion of 66 observations in the model allows for addressing the variations of a hospital across two successive years with certainty [72].

### Evaluating the impact of contextual factors on efficiency

Contextual factors which could influence the efficiency of a hospital (e.g., government regulations, geopolitical context, ..) are not under the control of the manager and can be accommodated in a DEA analysis [73]. The impact of environment on production was first considered by Charnes et al. [74]. The authors disentangled program efficiency from management efficiency by reference to empirical observations obtained from school programs.

Fried et al. [75] reviewed previous approaches to incorporating the external operating environment into a non-parametric measure of technical efficiency. Three categories classified by the applied method in the DEA literature are:

1. The frontier separation approach: can be implemented only for categorical factors and requires a priori selection of the most important contextual factor [74].
2. The all-in-one approach: Single-stage DEA estimation of the effects of contextual factors had been developed by Banker & Morey [76]. The procedure includes the external operating environment variables directly in the linear programming problem along with the traditional inputs and outputs. However, this approach requires that the external variable is classified as an input or an output in advance. Camanho et al. [77] propose a model that distinguishes between the influence of internal nondiscretionary factors and external nondiscretionary factors to estimate inefficiency.
3. The two-stage approach: The typical two-stage approach follows a first stage DEA estimation of efficiency based on inputs and outputs, then a second stage regression analysis seeking to explain variation in first stage efficiency scores concerning environmental factors. Some studies apply Ordinary Least Squares (OLS) regression to estimate the significant influence of contextual factors in the second stage; others use a Tobit regression model [78]. Ray [31, 79] was the first to apply the two-stage DEA model where the estimated efficiency scores in the first stage are regressed on contextual variables in the second stage.

Despite a large number of useful applications of the two-stage DEA method [29, 45, 80], it has been criticized and different examinations of the statistical consistency of the method provided contrast conclusions that call for further testing [15]. Banker & Natarajan [14] show by simulation that the two-DEA estimator for the contextual variables is statistically consistent when OLS or Maximum Likelihood Estimator (MLE) is applied in the second stage. This method requires the contextual factors to be independent of the input variables, but the contextual factors may be correlated with each other. Hoff [28] concluded that Tobit regression is sufficient to represent the second stage DEA models when compared with alternative methods or with the OLS. McDonald [27] came to a similar conclusion as Hoff, but he advocated not using Tobit regression.

Kieschnick & McCullough [81] recommended using parametric regression rather than using quasi-MLE unless the sample size is large enough to justify the argument underlying the quasi-MLE. Simar & Wilson [82] had sharply criticized the two-DEA method for lack of a coherent data generating process (DGP) and for the bias and serial correlation of the DEA efficiency estimates. They argue that the conventional methods of statistical inference are invalid in the second stage regression. Then, the authors propose the use of a bootstrap method to correct for the small sample bias and serial correlation of the DEA efficiency estimates. Later, Daraio, et al. [83] tested the assumptions required for two-stage estimation and rejected them in the non-parametric setting.

We follow Banker & Natarajan [14] and regress the DEA-CCR estimates of 66 observations during 2010–2015 on ten potential contextual factors. We run Tobit regression models to identify which environmental factors have a significant influence on the productive efficiency of Palestinian hospitals. The regression model has a censored structure because the dependent variable yielded from DEA-CCR model is limited between zero and one, while the independent variables that correspond to one can be observed. Then, Tobit regression which takes the censored structure into account is suggested. The model supposes that there is a latent dependent variable Y_p_^*^, this unobserved variable linearly depends on the independent variables X_p_ via a set of parameters βs. There is a normally distributed error term ε_p_ to capture random influences on the relation. The observed value of the dependent variable Yp (Eq. 4) is defined to equal the “latent variable” whenever the latent variable is above zero, and to equal “zero” otherwise, where:

4 $$ {\displaystyle \begin{array}{l}{Y}_p={Y}_p^{\ast}\kern0.5em if\kern0.5em {Y}_p^{\ast }>0\\ {}{Y}_p=0\kern0.5em if\kern0.5em {Y}_p^{\ast}\le 0\\ {}{Y}_P^{\ast }=\beta \kern0.5em {X}_p+{\varepsilon}_p\end{array}} $$

The CCR-DEA scores are right censored data, following the literature, the efficiency scores are transformed to become left-censored data of inefficiency scores the used Eq. 5 as suggested by Jon A Chilingerian [29, 33, 40]:

5 $$ Inefficiency\kern0.5em score=\left(\frac{1}{CCR\; DEA\; score}\right)-1 $$

To estimate the regression coefficients (βs), we applied the Maximum Likelihood Estimation (MLE) method in Tobit regression [45, 84]. Regression coefficients are interpreted in the same manner as the ordinary least-squares OLS regression. The only difference is the interpretation of the factor signs. Specifically, negative sign means better efficiency and positive sign means more inefficiency [33]. The initially estimated general model (Eq. 6) included all the proposed predictors:

6 $$ Ineffienciency={\beta}_0+{\beta}_1\kern0.5em BOR+{\beta}_2\kern0.5em OPIPR+{\beta}_3\kern0.5em ALOS+{\beta}_{4\kern0.5em }\kern0.5em ADHIR+{\beta}_{5\kern0.5em }\kern0.5em SIZE+{\beta}_6\kern0.5em LOC+{\beta}_7\kern0.5em REEP+{\beta}_8\kern0.5em HBFP+{\beta}_9\kern0.5em PRC+{\beta}_{10}\kern0.5em PPHB $$

We tested six Tobit regression models and selected the model with the highest value of the Log Likelihood (55.47 in model 6) to interpret our results (Table 5).

**Table 5:** Significant contextual factors: Tobit regression models

Predictors	model (1)	model (2)	model (3)	model (4)	model (5)	model (6)
BOR	−0.0171***	−0.0172***	−0.0170***	−0.0142***	−0.0149***	−0.0129***
	(0.00209)	(0.00203)	(0.00213)	(0.00221)	(0.00160)	(0.00229)
OPIPR	−0.218***	−0.211***	−0.219***	−0.207***	−0.204***	−0.197***
	(0.0588)	(0.0690)	(0.0426)	(0.0380)	(0.0485)	(0.0387)
ALOS	0.00596	0.00753	0.00553	−0.0302	−0.0391	−0.0626
	(0.0726)	(0.0663)	(0.0661)	(0.0469)	(0.0797)	(0.0808)
LOC	− 0.0543*	− 0.0526	− 0.0516	− 0.0345	− 0.0239	− 0.0117
	(0.0304)	(0.0365)	(0.0414)	(0.0387)	(0.0389)	(0.0381)
SIZE	0.0440	0.0413	0.0422	0.0467	0.0760*	0.0745*
	(0.0435)	(0.0375)	(0.0358)	(0.0408)	(0.0442)	(0.0389)
ADHR		−0.0432				
		(0.284)				
PEFP			0.000385			
			(0.00280)			
PPHB				0.00267**		0.00212
				(0.00134)		(0.00163)
PRC					0.0633***	0.0561***
					(0.0193)	(0.0216)
Constant	2.009***	2.019***	1.997***	1.558***	1.644***	1.327***
	(0.177)	(0.161)	(0.183)	(0.276)	(0.206)	(0.297)
Observations	66	66	66	66	66	66
Sigma	0.107	0.107	0.107	0.103	0.1	0.075
Wald X^2^	220.01	286.32	293.53	648.35	470.95	750.17
Prob. > X^2^	< 0.001	< 0.001	< 0.001	< 0.001	< 0.001	< 0.001
Pseudo R^2^	2.682	2.683	2.683	2.77	2.83	4.12
Log likelihood	38.14	38.15	38.15	40.15	41.45	55.47

Standard errors in parentheses

*** *p* < 0.01, ** *p* < 0.05, * *p* < 0.1

## Results

### The sample characteristics

We analyzed the efficiency 11 public hospitals operating in West Bank from 2010 to 2015. They utilize 1377 beds which make up 97.4% of the total public hospital beds in West Bank. It is worth noting the uneven spread of care providers across the governorates. (1) The distribution of public hospital beds per 10,000 citizens varies largely between seven beds and 27 beds. (2) The distribution of the primary healthcare centers per 10,000 citizens varies across governorates from 1.84 centers to 4.08 centers (3) The percentage of public hospital beds to the overall beds vary across governorates from 22 to 100% (see Table 2). The uneven distribution of providers is a remnant of previous political regimes in which the public sector evolved in West Bank [4]. Surprisingly, hospital P11 in Hebron governorate works at 120.4% occupancy rate. This hospital may show efficient scores, but it is reasonable to question the quality of care provided.

### Input-output variables

**Fig. 3:**
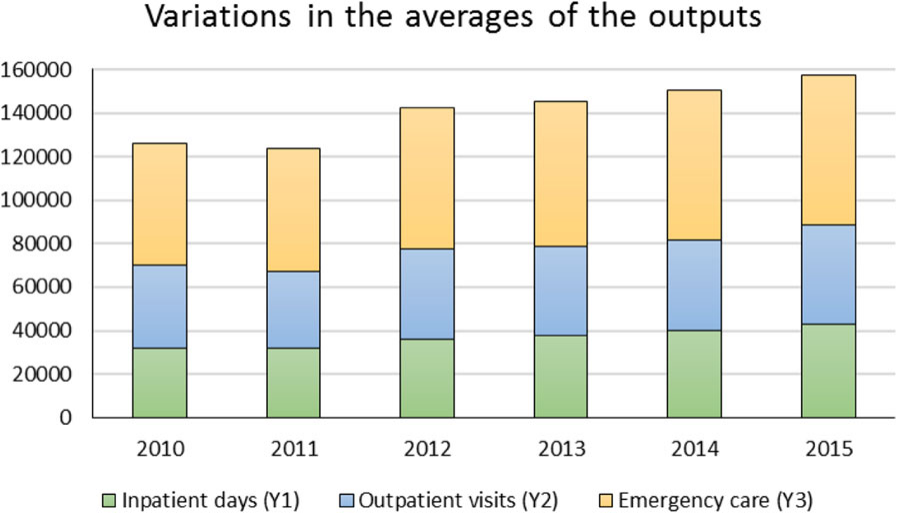
Year-Specific means of produced outputs

**Fig. 4:**
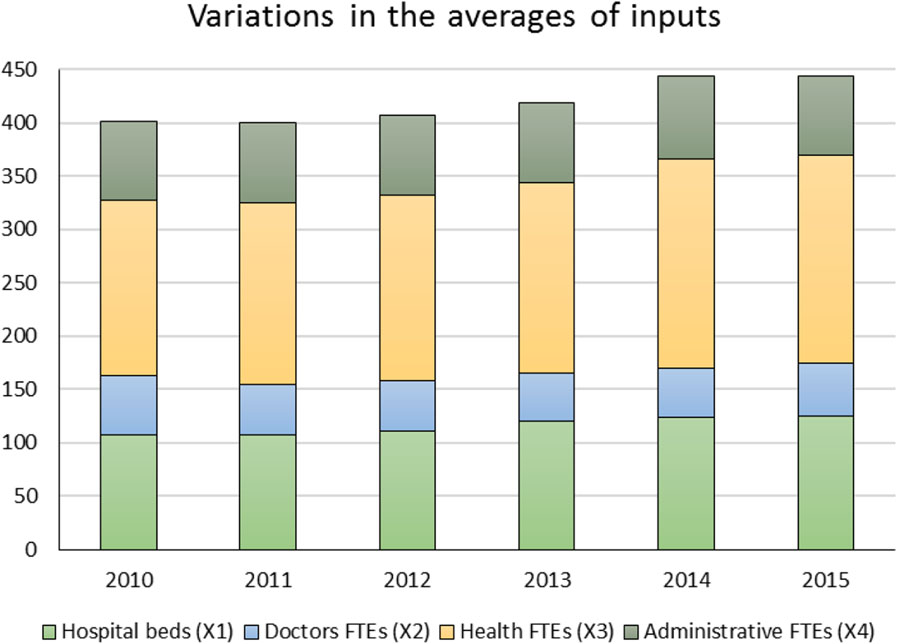
Year-specific means of consumed inputs

Figure 3 illustrates year-specific variations in means of the outputs produced during the study period. Since the year 2012, there is a consistent increase in emergency services that contributed significantly to the variations in the provided hospital services. However, input levels had slightly increased during the study periods, growth in the average number of health FTEs (e.g., nurses, laboratory technicians, radiology technicians) is noticed in Fig. 4.

### Hospitals’ efficiency, the year 2015

To make results more tangible for inefficient hospitals, we started analyzing the year 2015 to shape opportunities for potential improvements. Table 6 summarizes the individual efficiency scores for 11 hospitals in 2015. As estimated by the CCR model, under the Constant Returns to Scale (CRS) frontier, average efficiency was 0.86; scores ranged from 67 to 100%. As a result, the Palestinian public hospitals have the potential to reduce 14% of their inputs while producing the same levels of services.

**Table 6:** Efficiency estimates and Returns to Scale during 2015

Hospital	Beds	CCR (%)	Rank 1–11	BCC (%)	SE^*^ (%)	Σ λ CCR	Returns to Scale
P01	36	1	1	1	1	1	CRS
P02	50	0.67	11	1	0.67	0.53	IRS
P03	54	0.75	9	0.97	0.77	0.71	IRS
P04	55	1	1	1	1	1	CRS
P05	58	0.83	7	0.97	0.86	0.53	IRS
P06	117	0.85	6	0.85	1	1	CRS
P07	131	0.69	10	0.7	0.99	0.97	IRS
P08	163	0.92	4	0.95	0.97	1.22	DRS
P09	200	0.83	7	0.92	0.9	1.46	DRS
P10	238	0.87	5	1	0.87	2.06	DRS
P11	275	1	1	1	1	1	CRS
Average		0.86		0.94	0.91		

*SE, Scale efficiency

Three efficient hospitals P01, P04, and P011 constructed the best practices CCR frontier. These hospitals were efficient in the overall sense in both pure technical and scale efficiency. Hence, hospitals P01, P04, and P011 are operating under CRS. As for the other inefficient hospitals (Table 6), the sum of the optimal lambdas of the CCR model classifies each hospital as either operating under Increasing Returns to Scale (IRS) or Decreasing Returns to Scale (DRS). If Σλ > 1, then DRS prevail and if Σλ < 1, then IRS prevail [67, 68].

As estimated by the DEA-BCC model, under variable returns to scale approach, five hospitals P01, P02, P04, P10, and P11were pure technically efficient. The others, P03, P05, P06, P07, P08, and P09 suffer pure technical inefficiency and can individually reduce their inputs by (1- BCC score) without reducing the levels of the provided outputs. Hospital P06 is scale efficient, and its inefficient performance is attributed to technical inefficiency as illustrated by the BCC score 85% implying a potential savings of 15% of inputs producing the same level of outputs.

Scale efficiency measures show hospitals P01, P04, and P11 as efficient and working under constant returns to scale. Four hospitals P02, P03, P05, and P07 were operating under increasing returns to scale. Their average scale efficiency is 82.2% and could theoretically increase their size by 17.3% to achieve optimal scale. Hospitals P08, P09, and P10 were operating under decreasing returns to scale implying that their service outputs increase by a smaller proportion compared to an increase in inputs. Their average scale efficiency is 91.4% and appears to be higher than the average for increasing returns to scale hospitals. Thus the effect on scale efficiency due to the size of the hospital is stronger on small hospitals than on large ones. Managers of large hospitals P08, P09, and P10 are weakly controlling their service operations. Better adaptation to new technologies will help them improve their scale efficiency [41, 85].

### Hospitals’ efficiency, year-specific analysis

Table 7 summarizes year-specific efficiency estimated by the DEA-CCR and the DEA-BCC frontiers of 66 observations. During the whole study period, the average CCR efficiency score was 85.29% in the overall sense of technical efficiency. To become efficient, the public hospitals in Palestine could save 15% of their resources to produce the given level of the observed outputs. The average pure technical efficiency as given by the BCC e scores was 93.7%, managerial practices could enhance performance by 6.3%. The average scale efficiency was 91%, resource allocation can enhance scale efficiency. Savings will have a significant impact on hospital operations and on reducing the volume of purchased hospital services and save public money.

**Table 7:** Overall efficiency TE and pure technical efficiency PTE during 2010–2015

Hosp.	Beds	CCR scores (TE)						BCC scores (PTE)					
		2010	2011	2012	2013	2014	2015	2010	2011	2012	2013	2014	2015
P01	36	1	1	1	1	1	1	1	1	1	1	1	1
P02	50	0.64	1	0.72	0.66	0.6	0.67	0.98	1	0.99	0.95	0.99	1
P03	54	0.53	0.6	0.72	0.72	0.67	0.75	0.84	0.81	0.95	0.92	0.9	0.97
P04	55	1	1	1	1	1	1	1	1	1	1	1	1
P05	58	0.93	0.99	0.78	0.91	0.81	0.83	1	1	0.94	1	0.95	0.97
P06	117	0.75	0.91	0.79	0.82	0.75	0.85	0.76	0.94	0.83	0.83	0.8	0.85
P07	131	0.84	0.8	0.89	0.86	0.69	0.69	1	0.95	0.93	0.87	0.73	0.7
P08	163	0.9	1	0.83	0.73	0.74	0.92	0.93	1	0.84	0.82	0.84	0.95
P09	200	0.69	0.82	0.79	0.87	0.86	0.83	0.75	0.83	0.79	0.94	0.88	0.92
P10	238	0.72	0.83	0.86	1	0.86	0.87	1	1	1	1	1	1
P11	275	1	1	1	1	1	1	1	1	1	1	1	1
Average %		81.8	90.5	85.3	87.0	81.6	85.5	93.3	95.7	93.4	93.9	91.7	94.2
On the frontier		3	5	3	4	3	3	6	7	4	5	4	5
Percentage %		27	46	27	36	27	27	55	64	36	46	36	46

Scale efficiency score (SE) = CCR score / BCC score

**Fig. 5:**
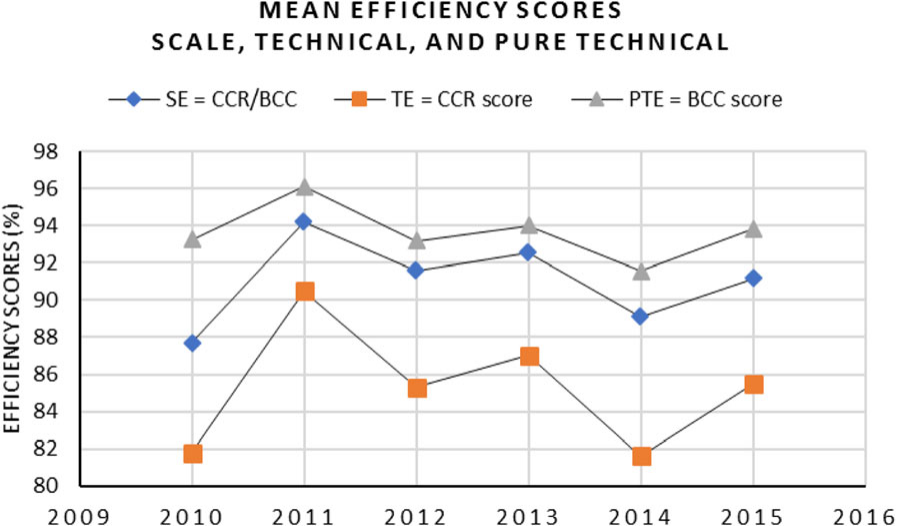
Year-specific mean efficiency scores; Technical Efficiencies (TE), Scale Efficiencies (SE), and Pure Technical Efficiencies (PTE)

During 2011, hospitals show the best performance level as given by average pure technical efficiency of 90.5% (see Fig. 5). The years 2010 and 2014 show relatively poor performance as given by the average efficiencies of 81.8 and 81.6% respectively. Hospitals, in the general sense, gave the idea as improving their performance during the study period. More specifically, there was 4% increase in the average efficiency scores in 2015 compared with 2010.

### The impact of environmental factors on hospitals’ efficiency

We included the 66 observations from the years 2010 to 2015 in simultaneous CCR estimates (see Additional files 1 and 2). The resulting cross-sectional data is right-censored then transformed to left censored inefficiency scores. Then, we regressed the inefficiency scores on the proposed contextual factors in Table 3. To get more robust results, we tested six Tobit models using Stata 14 to examine the significance and to estimate the parameters. We started with four predictors to get a significant overall model, then included additional predictor in each model, model 6 showed four significant predictors. We run robust standard error procedure to avoid the heteroscedasticity; Table 5 presents the results of Tobit regressions.

The joint null hypothesis as H_0_: β1 = β2 = β3 = β4 = β5 = β6 = β7 = β8 = β9 = β10 = 0 is rejected at the 1% level of significance when given at least one non-zero parameter in all the models. The final empirical regression model (Table 5) including the significant predictors at 10% significance level was (model 6):

7 $$ Inefficiency={\beta}_0+{\beta}_1\kern0.5em BOR+{\beta}_2\kern0.5em OPIPR+{\beta}_5\kern0.5em SIZE+{\beta}_9\kern0.5em PRC $$

## Discussion

### Hospitals’ efficiency

When decomposing the overall efficiency into pure technical and scale efficiency components, it is worth noting five findings:

1. There are different sources of inefficient performance, inefficient operations of P02 and P10 are attributed to scale effect while inefficient operations of P06 and P07 are attributed to pure technical effect. P02 and P10 represent a ministry problem while P06 and P07 represent a hospital management problem.
2. Hospital P02 is 67% scale efficient while 100% pure technically efficient and was operating under increasing returns to scale suggesting an increase in its size to catch optimal production level.
3. As expected, hospital P10 is 87% scale efficient while 100% pure technically efficient and was operating under decreasing returns to scale, a state of lack of control over resources. Because P10 is a hospital in Ramallah titled “The Medical Complex” resulted from the previous merging of three hospitals in the governorate, this may explain inefficiency and a certain level of managerial failure or lack of control.
4. P07 and P02 were ranked in 10 and 11 positions respectively, more efforts should be focused on their performance improvement.

### Significant contextual factors

Four predictors were significant in explaining hospital inefficiency, they are: (1) the bed occupancy rate (BOR) at 1% significance level *p* <  0.01. (2) the outpatient visits to inpatient days ratio (OPIPR) at 1% significance level *p* <  0.01. (3) the hospital size (SIZE) at 10% significance level *p* <  0.1. (4) and the available number of primary healthcare per 10,000 citizens (PRC) living in the served governorate at 1% significance level *p* <  0.01.

The coefficients of BOR and OPIPR are negative implying that increasing these predictors by one unit will lead a decrease in the predicted hospital inefficiency by 0.0129 and by 0.197 respectively, holding all other variables in the model constant. The higher the hospital BOR or OPIPR, the lower the predicted inefficiency score with a substantial effect of OPIPR, a change of one unit in OPIPR will decrease inefficiency level by 19.7%. Our result was expected and goes consistently with the previous evidence [40, 42].

The coefficient of SIZE was positive implying that inefficiency and SIZE change in the same direction. Results indicate that predicted inefficiency for large hospitals is 0.0745 higher than for small hospital keeping all other predictors constant. As hospital capacity increases for more than 130 beds, hospital inefficiency is more likely to increase. Our results are inconsistent with previous evidence from Turkey [86].

The coefficient of PRC was positive implying that increasing the number of available primary centers per 10,000 by one unit in a governorate will lead an increase in the predicted inefficiency score by 1.327. The more available primary healthcare centers in the served region, the less efficient the hospital working there. This result may be explained as the more primary healthcare centers; healthcare seekers have more flexibility in choosing a provider and become less likely to go to a public hospital [87].

## Conclusions

Using data from 2010 through 2015, we assessed the technical efficiency of public hospitals in West Bank with the aim of identifying potential improvements. The research work has the privilege to present the first investigation of the productive efficiency of hospital services in Palestine. Additionally, this work originates as reference study for future research within the distinct country conditions of the Occupied Palestinian Territories. The analysis builds on a set of variables that conform with nature of services in the Palestinian public hospitals being a general type of hospitalization. The output measures conceptualize the volume of benefits delivered to the public regarding, inpatient days, outpatient visits, and emergency services. The input measures considered the hospital size and personnel structure (capital and labor). Furthermore, this study took the basic data envelopment analysis (DEA) further by employing the second stage DEA analysis. The work incorporates the environmental factors that influence the inefficient performance of hospitals.

The generated information of this research provides valuable insights to hospital managers on their operational practices, as well as, to policymakers at the ministry level whose decisions may influence the significant contextual factors. The main findings of this study are: (1) The potential savings of 14.5% of resource consumption without reducing the volume of the provided services. (2) The strong effect of outpatient-inpatient ratio on efficient performance. A one-unit increase in OPIPR will lead a decrease of 19.7% in the predicted inefficiency level, holding all other factors constant. The synergic practices between inpatient and outpatient units is a predictor of efficient managerial behavior to better allocation of input resources [40].

The first aim of this study was to evaluate the productive efficiency of the Palestinian public hospitals in West Bank. The empirical results show that during the year 2015, three hospitals were technically efficient in the overall sense (DEA-CCR), and they were more successful to transform the constrained inputs into hospital services. The relative efficiency of other hospitals ranged from 67 to 92%, and the average efficiency of all the observed hospitals was 85.5% and can save 14.5% of their resources. Further analysis of the DEA-CCR and the DEA-BCC scores allowed us to decompose the inefficient components of hospital operations into pure technical and scale efficiencies. Inefficient operations of P06 and P07 are attributed to managerial effects only as given by the scale-free BCC efficiencies 85 and 70% respectively. However, the inefficient operations of hospitals P02 and P10 are attributed to scale effects. Moreover, the year 2011 exhibited a best average level of performance, and the year 2014 exhibited the poorest average performance level. During the study period, the average scale efficiency as given 91% level had a larger effect on driving the given overall inefficiency than the average pure technical efficiency as given 93.7% level.

Therefore, findings show the need for revision of the resource allocation policies. Hospitals working under increasing returns to scale can share additional workload to reach their optimal size of operations, considering this opportunity. Findings also suggest the need for revision of the criteria for making a referral decision to purchase hospital services. Finally, the managers of inefficient hospitals can direct and quantify their efforts to benchmark best performer.

The second aim of this study was to explore the determinants of inefficient performance among the Palestinian public hospitals. Tobit regression models identified four significant relations between working conditions and operational inefficiency levels. Bed occupancy rate has a negative impact on inefficiency levels because hospitals with higher occupancy rates will take advantage of full bed capacity, findings suggest setting a proper level of health personnel following bed occupancy rates will enhance efficiency [11, 88]. Outpatient visits as a proportion of inpatient days had a negative impact on inefficiency [40]. Since outpatient services require less personnel, managing this ratio while considering for case mix will help managers make better use of resources.

The other three factors: (1) the size of the hospital; (2) the number of available primary care centers within the governorate. These factors were positively related to inefficient performance. Setting the appropriate combination of healthcare providers in a particular region in Palestine is within the role of the Ministry of health. Evidence from Turkey revealed opposite effect of size on hospital performance [86]. Capacity plans that consider the volume, type and ownership following population health needs will reduce the impact of the prevalent weak structure in country’s hospital settings. Finally, hospital location in Northern governorates was negatively related to inefficiency. Previous evidence showed the impact of location on hospital efficiency due to demographic and socioeconomic differences [54]. Managing these factors will guide policy makers at the Ministry level for appropriate policies and regulations towards better efficient operations among the Palestinian public hospitals.

Although the study was the first to examine the productivity of Palestinian public hospitals, it has some important limitations. First, the study excluded hospitals working in Gaza and results can’t be secured at the country level. Due to the relative nature of DEA, the exclusion of hospitals in Gaza could make our results overestimated. Second, due to lack of data, the study didn’t include operating costs. Third, the small sample size makes the results very sensitive to measurement error. However other studies used small sample size, it is the reality when the examined group of DMUs is small [89]. Like many developing countries, because of lack of data associated with qualitative information, the scope of our study was limited to examine decisions made by managers and ministry administrators in respect of the quantity and the distribution of resources. Future research could extend the results of this study in many ways:. Research may consider the clinical quality of services and patient satisfaction.

Finally, the study uncovered hospitals working at over occupancy rate (> 100%), more specific, the largest hospital P11 with 275 beds. While this hospital displayed efficient performance, quality is questioned and requires more investigations to examine whether the adoption of policies of over occupation in some Palestinian hospitals leads to premature discharges or other medical complications.

## Additional files

Additional file 1: Right-censored data; efficiency of 66 observations given by the DEA-CCR estimates. (DOCX 32 kb)

Additional file 2: Left-censored data; inefficiency scores of 66 observations. (DOCX 33 kb)

## Abbreviations

2-DEA: Two-stage data envelopment analysis
BCC: a model developed by Banker Charnes and Cooper 1984
CCR: a model developed by Charnes, Cooper, and Rhodes 1978
CRS: Constant returns to scale
DEA: Data envelopment analysis
DMUs: Decision making units
DRS: Decreasing returns to scale
FTE: Full time employees
GDP: Gross domestic product
GS: Gaza strip
IRS: Increasing returns to scale
MLE: Maximum likelihood estimation
NGO: Non-Governmental Organization
OECD: Organization for Economic Cooperation and Development
OLS: Ordinary least squares
OPT: Occupied Palestinian Territories
PA: Palestinian authority
PCBS: Palestinian Central Bureau of Statistics
PMMS: Palestinian military medical services
PMoH: Palestinian ministry of health
PTE: Pure technical efficiency
SE: Scale efficiency
TE: Technical efficiency
UNRWA: United Nations Relief and Works Agency for Palestine Refugees
VRS: Variable returns to scale
WB: West Bank
WHO: World Health Organization
