# Supplementary material for: Measuring the efficiency of Palestinian public hospitals during 2010–2015: an application of a two-stage DEA method
Source: BMC Health Serv Res. 2018 May 29;18:381. doi: 10.1186/s12913-018-3228-1 (PMC5975658; doi:10.1186/s12913-018-3228-1)
Supplement: Supplementary file 1 — Right-censored data; efficiency of 66 observations given by the DEA-CCR estimates. (DOCX 32 kb) [file 12913_2018_3228_MOESM1_ESM.docx]

# Measuring the efficiency of Palestinian public hospitals during 2010-2015: An application of a two-stage DEA method

## Additional file 1

Right-censored data; efficiency of 66 observations given by the DEA-CCR estimates.

| **Hospital** | CCR efficiency: Simultaneous estimation of 66 observation | | | | | |
| --- | --- | --- | --- | --- | --- | --- |
|  | 2010 | 2011 | 2012 | 2013 | 2014 | 2015 |
| P01 | 1 | 0.92 | 1 | 0.89 | 0.98 | 0.97 |
| P02 | 0.57 | 0.66 | 0.67 | 0.57 | 0.58 | 0.64 |
| P03 | 0.49 | 0.51 | 0.67 | 0.63 | 0.6 | 0.65 |
| P04 | 0.97 | 0.93 | 0.99 | 1 | 1 | 0.98 |
| P05 | 0.88 | 0.68 | 0.71 | 0.78 | 0.77 | 0.82 |
| P06 | 0.62 | 0.63 | 0.69 | 0.72 | 0.69 | 0.69 |
| P07 | 0.73 | 0.72 | 0.82 | 0.73 | 0.65 | 0.68 |
| P08 | 0.75 | 0.78 | 0.77 | 0.65 | 0.71 | 0.78 |
| P09 | 0.64 | 0.64 | 0.76 | 0.76 | 0.75 | 0.78 |
| P10 | 0.64 | 0.74 | 0.8 | 0.88 | 0.79 | 0.84 |
| P11 | 1 | 1 | 1 | 1 | 1 | 1 |
| Average | 0.75 | 0.75 | 0.81 | 0.78 | 0.77 | 0.80 |
