# Supplementary material for: Measuring the efficiency of Palestinian public hospitals during 2010–2015: an application of a two-stage DEA method
Source: BMC Health Serv Res. 2018 May 29;18:381. doi: 10.1186/s12913-018-3228-1 (PMC5975658; doi:10.1186/s12913-018-3228-1)
Supplement: Supplementary file 2 — Left-censored data; inefficiency scores of 66 observations. (DOCX 33 kb) [file 12913_2018_3228_MOESM2_ESM.docx]

# Measuring the efficiency of Palestinian public hospitals during 2010-2015: An application of a two-stage DEA method

## Additional file 2

Left-censored data; inefficiency scores of 66 observations.

$$Inefficiency score=\left( \frac{1}{CCR DEA score} \right)-1$$

| **Hospital** | Transformed inefficiency: Simultaneous estimation of 66 observation | | | | | |
| --- | --- | --- | --- | --- | --- | --- |
|  | 2010 | 2011 | 2012 | 2013 | 2014 | 2015 |
| P01 | 0.00 | 0.09 | 0.00 | 0.12 | 0.02 | 0.03 |
| P02 | 0.75 | 0.52 | 0.49 | 0.75 | 0.72 | 0.56 |
| P03 | 1.04 | 0.96 | 0.49 | 0.59 | 0.67 | 0.54 |
| P04 | 0.03 | 0.08 | 0.01 | 0.00 | 0.00 | 0.02 |
| P05 | 0.14 | 0.47 | 0.41 | 0.28 | 0.30 | 0.22 |
| P06 | 0.61 | 0.59 | 0.45 | 0.39 | 0.45 | 0.45 |
| P07 | 0.37 | 0.39 | 0.22 | 0.37 | 0.54 | 0.47 |
| P08 | 0.33 | 0.28 | 0.30 | 0.54 | 0.41 | 0.28 |
| P09 | 0.56 | 0.56 | 0.32 | 0.32 | 0.33 | 0.28 |
| P10 | 0.56 | 0.35 | 0.25 | 0.14 | 0.27 | 0.19 |
| P11 | 0.00 | 0.00 | 0.00 | 0.00 | 0.00 | 0.00 |
| *Transformed inefficiency scores = [(1/CCR efficiency) -1] | | | | | | |
